# A team science approach to discover novel targets for infantile spasms (IS)

**DOI:** 10.1002/epi4.12441

**Published:** 2020-12-22

**Authors:** The CURE Infantile Spasms Consortium, CURE Staff, Annamaria Vezzani, Anne T Berg, Daniel H Lowenstein, Henrik Klitgaard, Howard P Goodkin, Jong M Rho, Anna Maria Katsarou, Antonella Pirone, Aristea S Galanopoulou, Chris Dulla, Douglas R Nordli, Dumitru A Iacobas, Elliott Sherr, James Cloyd, Jana Veliskova, Jeff L Noebels, John J Millichap, John W Swann, Sookyung Koh, Libor Velisek, Lisa Coles, Manisha Patel, Meagan S Siehr, Michele Jacob, Ryan Seo, Svenja Heischmann, Solomon L Moshe, Tamar Chachua, Tufikameni Brima, Wenzhu B. Mowrey, H Steve White, Julie Milder, Laura Lubbers, Sloka S. Iyengar

**Affiliations:** ^1^ Citizens United for Research in Epilepsy (CURE) Chicago IL USA; ^2^ Prairie View A&M University Personalized Genomics Laboratory Prairie View TX USA

**Keywords:** Citizens United for Research in Epilepsy (CURE), Hypsarhythmia, infantile spasms, team science, West syndrome

## Abstract

Infantile spasms (IS) is a devastating epilepsy syndrome that typically begins in the first year of life. Symptoms consist of stereotypical spasms, developmental delay, and electroencephalogram (EEG) that may demonstrate Hypsarhythmia. Current therapeutic approaches are not always effective, and there is no reliable way to predict which patient will respond to therapy. Given this disorder's complexity and the potential impact of a disease‐modifying approach, Citizens United for Research in Epilepsy (CURE) employed a “team science” approach to advance the understanding of IS pathology and explore therapeutic modalities that might lead to the development of new ways to potentially prevent spasms and Hypsarhythmia. This approach was a first‐of‐its‐kind collaborative initiative in epilepsy. The IS initiative funded 8 investigative teams over the course of 1‐3 years. Projects included the following: discovery on the basic biology of IS, discovery of novel therapeutic targets, cross‐validation of targets, discovery of biomarkers, and prognosis and treatment of IS. The combined efforts of a strong investigative team led to numerous advances in understanding the neural pathways underlying IS, testing of small molecules in preclinical models of IS and generated preliminary data on potential biomarkers. Thus far, the initiative has resulted in over 19 publications and subsequent funding for several investigators. Investigators reported that the IS initiative generally affected their research positively due to its collaborative and iterative nature. It also provided a unique opportunity to mentor junior investigators with an interest in translational research. Learnings included the need for a dedicated project manager and more transparent and real‐time communication with investigators. The CURE IS initiative represents a unique approach to fund scientific discoveries on epilepsy. It brought together an interdisciplinary group of investigators—who otherwise would not have collaborated—to find transformative therapies for IS. Learnings from this initiative are being utilized for subsequent initiatives at CURE.

**Key points**

- Infantile spasms (IS) is a devastating epilepsy syndrome characterized by stereotypical spasms, developmental delay, and may be associated with Hypsarhythmia.
- Citizens United for Research in Epilepsy (CURE) funds research that could potentially transform the treatment landscape of epilepsy.
- Given the wealth of knowledge regarding IS, the animal models used for IS research, and the potential impact of a disease‐modifying treatment, CURE chose IS to focus its energies on, and developed the IS initiative.
- The initiative accelerated the pace of IS research, developing collaborations between investigators who otherwise perhaps would not have worked together, and trained junior epilepsy researchers.
- The IS initiative was the first‐of‐its‐kind research project aimed at driving epilepsy research, and learnings from the initiative are currently being used for additional research initiatives at CURE.

## 1 INTRODUCTION

### 1.1 Infantile spasms

In 1841, physician William West wrote a letter to the editor of the Lancet describing a “peculiar form of infantile convulsions” that he had witnessed in his son.^1^, ^2^ These paroxysms commenced at 4 months of age as a slight bobbing of the head that, over time, evolved in frequency and severity as to cause a complete heaving of the head forward toward his knees, and then immediately relaxing into the upright position. These episodes would be repeated at “intervals of a few seconds and repeated from ten to twenty or more times at each attack, each attack would not continue more than 2 or 3 minutes; he sometimes has two, three, or more attacks in a day.” These attacks would later be called Infantile spasms (IS) and the syndrome of IS, a triad of spasms, developmental delay, and the electroencephalographic (EEG) finding of Hypsarhythmia, would be labeled West syndrome.^3^, ^4^

Onset of IS is typically between 3 and 10 months of age. Children who experience IS may have other seizure types, and some may evolve to meet criteria for Lennox‐Gastaut syndrome. Estimates for incidence of IS range from 0.249 to 0.323 cases per 1000 live births^5^, ^6^, and incidence varies as a function of acquired insults such as brain damage.^7^ Other causes of IS are many, and range from CNS infection, to abnormalities in brain development, neurocutaneous syndromes and metabolic disorders.^8^ Genetic causes, when identifiable, tend to be heterogeneous, but informative for diagnosis and management of individuals.^9^

Diagnosis is confirmed by an ictal EEG recording, coincident with the spasm, in which an electrodecremental response occurs. In the inter‐ictal period, children with West syndrome may demonstrate Hypsarhythmia (a high‐voltage, disorganized, asynchronous EEG background with multifocal paroxysmal epileptiform discharges), although a small percentage may not.^10^ Etiologic diagnosis is made with the help of patient history, physical and neurological examination, and magnetic resonance imaging of the brain. Given its high diagnostic yield, genetic analysis has largely replaced previous metabolic testing.^11^, ^12^

**FIGURE 1:**
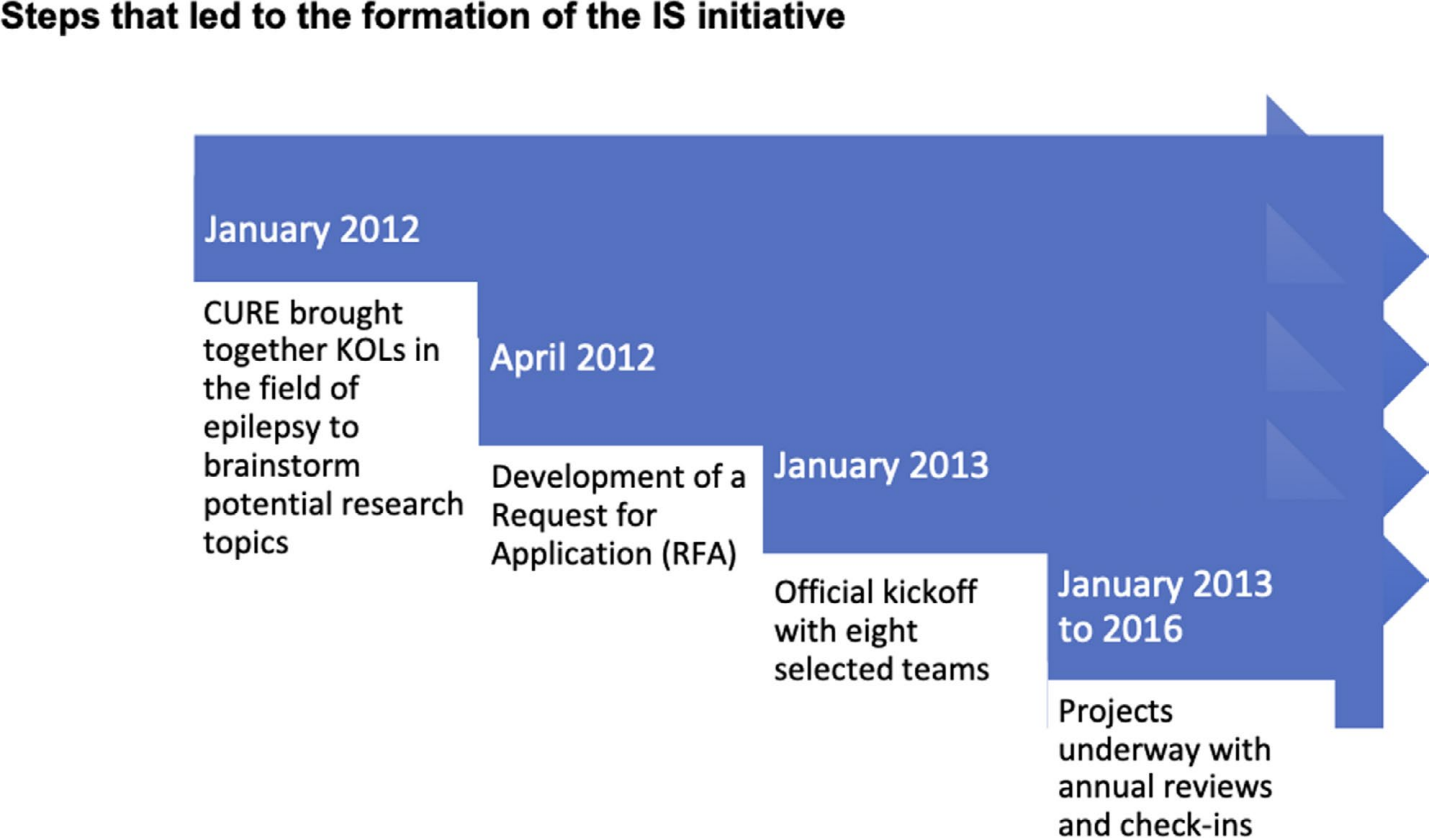
Steps that led to the formation of the IS initiative

Initial standard treatment for IS remains either hormonal therapy [eg, adrenocorticotropic hormone (ACTH), prednisone] or the anti‐seizure medication vigabatrin—this combination is effective in approximately half of the patients with IS.^13^, ^14^ Early control of spasms can lead to improved long‐term outcomes^15^; however, current treatments are not always effective and often have adverse side‐effects. Ongoing IS can lead to profound developmental delay, underscoring an additional unmet need in the field.^16^ More importantly, there is no reliable means to identify which patient will respond to which therapy. Thus, there is a clear need for expanding the understanding of IS pathophysiology and the development of more effective treatment strategies. Given the substantial knowledge about its molecular and genetic underpinnings, further research into IS could lead to transformative therapies (Figure 1).

### 1.2 Team science

The National Cancer Institute (NCI) defines Team Science Initiatives as “collaborative efforts to address a scientific challenge that leverages the strengths and expertise of professionals trained in different fields.” The team science approach is particularly appropriate for complex problems with numerous interacting variables, as it has the potential to produce significant scientific outcomes, and a broader, more rapid dissemination of scientific findings.^17^, ^18^, ^19^, ^20^, ^21^ For example, the Allen Institute for Brain Science spearheaded the Allen Brain Observatory, the goal of which is to record the activity of millions of neurons in specific areas of the mouse brain as the mouse engages in certain behaviors. This required the concerted effort of many professionals.^22^ The US Brain Research through Advancing Innovative Neurotechnologies (BRAIN) Initiative^23^, and the Next Generation Networks for Neuroscience (NeuroNex)^24^ are collaborative efforts developed to uncover physiological workings of the brain. However, to our knowledge, prior to 2012, there had not been an organized, integrated, multi‐disciplinary effort where epilepsy research was guided in real‐time.

### 1.3 Citizens United for Research in Epilepsy (CURE)

Founded in 1998, CURE’s mission is to cure epilepsy by funding and promoting patient‐focused research. CURE identifies cutting‐edge research and challenges scientists worldwide to collaborate and innovate in pursuit of a cure. CURE has been instrumental in funding investigator‐initiated, basic science grants that have advanced knowledge of the underlying causes of pediatric and acquired epilepsies and Sudden Unexpected Death in Epilepsy.^25^

## 2 METHODS

### 2.1 Formation of the IS initiative

In January 2012, CURE assembled an advisory panel of epilepsy key opinion leaders (KOLs) to brainstorm potential topics and therapeutic areas on which to focus research. The advisors were experts in the fields of epidemiology, adult and pediatric epilepsy, epilepsy genetics, ketogenic diet, biomarkers, inflammation/immune response in epilepsy, and drug discovery and development. The question posed was “If CURE were to allocate a significant investment of 3‐5 million dollars over 3 years to one specific epilepsy, what would that be, and what would be the approach to advance a transformative therapy?”

Day 1 of the workshop consisted of discussion focused on epilepsy epidemiology, genetic epilepsies, acquired epilepsies in adults and pediatric epilepsies. KOLs were challenged to identify an unmet need that could, with appropriate resources, be prevented, cured, or modified by the development of a transformative therapy. Of the topics discussed, it was felt that advancing a greater understanding of IS could lead to the identification and development of a new therapy for this often‐devastating pediatric encephalopathy. Day 2 focused on IS and considered a number of factors including: clinical issues surrounding diagnosis and treatment, the efficacy and adverse effects of current treatments, availability of biomarkers and issues such as predicting which patient would benefit from a specific therapy.

IS was selected as the subject of CURE’s initiative for the following reasons:

- Since current treatment options do not control spasms in all patients, discovery of a novel therapeutic target for IS could be revolutionary.
- Given a lack of validated biomarkers that could predict which patients will respond best to treatment, the initiative could lead to the discovery of an IS biomarker that could transform the treatment landscape for IS.
- A well‐described clinical and electrophysiological phenotype as a surrogate marker could be used to assess treatment intervention and target engagement.
- Early and effective treatment could prevent cognitive and developmental delays.^26^
- There are several animal models that could be used for therapy screening ^27^, ^28^, ^29^, ^30^, ^31^ and cross‐validation. Animal models that replicate key aspects of the chronic or evolving course of human IS play an important role in drug discovery. Genetic animal models include aristaless‐related homeobox gene (Arx) Knock‐In (Arx(^GCG)10+7^) model, Arx Conditional Knock‐Out (cKO) model, adenomatous polyposis coli (APC) cKO model, and the tuberous sclerosis complex (TSC) models in mice. Acquired models include the tetrodotoxin (TTX) rat model, prenatal betamethasone‐postnatal N‐Methyl‐d‐aspartate (NMDA) model, and the multiple‐hit rat model.^27^ The multitude of IS animal models enabled the opportunity to test a potential target across multiple models. The steps that led to the formation of the IS initiative are shown in Figure 1.

### 2.2 Structure of the IS initiative

The KOL advisory panel, in conjunction with the CURE scientific team developed a Request for Applications that was released in April 2012 for anticipated funding to begin in January 2013. After a review of 27 Letters of Intent, 11 full proposals were invited and reviewed. Of these, 8 proposals were recommended for funding (Table 1). The primary criterion for selection was that a proposal had to contribute to the interdisciplinary approach of the IS initiative. Another criterion was that a proposal needed to bring in broad views on IS treatment, for example, genetics, neuronal networks, basic neurobiology of IS. In January 2013, the 8 Principal Investigators (PI) were invited to present their proposals to the entire group of investigators, the CURE scientific team, and the advisory panel, to critique hypotheses and specific aims. Following this review, aims, budgets, and milestones were revisited by the CURE scientific team and the advisory panel to set investigators’ expectations. The goal of the project was to develop a scientifically sound collaborative, team‐based approach where the investigative teams critiqued each other to establish focused, goal‐driven scientific aims and thereby, “*let the science drive the direction.”*

**TABLE 1:** Hypotheses and specific aims of projects funded as part of the infantile spasms (IS) initiative

Investigator, Institution	Title	General description
Chris Dulla, Tufts University	APC (adenomatous polyposis coli) cKO mouse as a new model of infantile spasms	To study conditional loss of adenomatous polyposis coli (APC) in excitatory neurons, ß‐catenin, and the role of aberrant Wnt and synaptogenic protein networks in synaptic and circuit dysfunction associated with IS.
Aristea Galanopoulou, Albert Einstein College of Medicine	Identifying new therapies for infantile spasms	Preclinical screening for new treatments in two chronic models of chronic IS.
Douglas Nordli, Lurie Children's Hospital	Prevention of West syndrome	To study the EEG pattern of pre‐Hypsarhythmia and its prediction of West syndrome. To test whether ACTH improves the EEG in infants with pre‐Hypsarhythmia and decreases the likelihood of progression to West syndrome.
John Swann, Baylor College of Medicine	Infantile Spasms: Mechanisms and Consequences as Therapeutic Targets	To identify neurophysiological biomarkers that can be used to predict the response to a drug in terms of cessation of IS. To determine the effects of eliminating spasms on development of focal epilepsy and impaired learning and memory later in life. To identify novel therapeutic strategies and analogs to stop spasms and to ameliorate the underlying neurodevelopmental abnormalities.
Manisha Patel, University of Colorado Denver	Metabolomic Biomarker Discovery in Infantile Spasms	To study the metabolome for potential biomarkers for IS.
Jeffrey Noebels, Baylor College of Medicine	Infantile Spasms syndrome: From gene to bedside, an accelerated path to new therapy	To study the effects of sex hormones in the complex neurological phenotype of IS. To investigate whether seizures were caused by the failure of interneuron migration and maturation in the developing brain.
Elliott Sherr, University of California San Francisco^a^	Infantile Spasms: Clinical and Genetic Predictors of Outcomes and Therapeutic Insights	To study mutations in specific genes and their effect on neurodevelopmental abnormalities in IS. To study infantile spasms of unknown cause (UCIS).
Libor Velíšek, New York Medical College^a^	Developing and testing novel treatments for infantile spasms	To study the role of hypothalamus‐linked peptides as therapeutic tools for IS.

^a^ Projects that were discontinued during the IS initiative

The role of the advisory panel was to oversee projects, advise the teams regarding their progress and provide scientific insight. The advisory panel attended meetings to review scientific direction and served in a consultative capacity to CURE staff and to the scientific teams. The advisory panel assisted CURE staff in interpreting data and assessing how the outcomes of specific projects aligned with the overall goals of the initiative, ensured transparency, and saw to it that questions were being asked and answered openly. The advisory panel also met separately with the CURE scientific staff to discuss progress and develop additional milestones.

While the advisors had a consultative role, the CURE scientific team performed the operational and project management tasks of the initiative. The team reviewed and advised on revision of specific aims of projects and communicated directional shifts in aims to the investigators. In addition, they negotiated revised budgets and made recommendations to the CURE Board of Directors on financial matters.

**TABLE 2:** List of publications, abstracts, grants, external funding, and patents as a result of CURE IS initiative

Discovery on the basic biology of IS
**Noebels laboratory**
*Publications* 1. Siehr M, Massey C, Noebels JN. Arx expansion mutation perturbs cortical development by augmenting apoptosis without activating innate immunity in a mouse model of X‐Linked Infantile Spasms Syndrome. Dis Model Mech. 2020, pii: dmm.042515. 2. Chen C, Sgritta M, Mays J, et al Therapeutic inhibition of mTORC2 rescues the behavioral and neurophysiological rescues the behavioral and neurophysiological abnormalities associated with Pten‐deficiency. Nat Med 2020; 25 (11): 1684‐1690.
**Dulla laboratory**
*Publications* 1. Dulla CG. Utilizing Animal Models of Infantile Spasms. Epilepsy Curr. 2018;18(2):107‐112. 2. Pirone A, Alexander JM, Lau LA, et al APC conditional knock‐out mouse is a model of infantile spasms with elevated neuronal β‐catenin levels, neonatal spasms, and chronic seizures. Neurobiol Dis. 2017; 98:149‐157. 3. Pirone A, Alexander JM, Koenig JB, et al Social stimulus causes aberrant activation of the medial prefrontal cortex in a mouse model with autism‐like behaviors. Front Synaptic Neurosci. 2018; 10:35.
*Grant* NINDS R01; The role of beta‐catenin in the pathophysiology of infantile spasms: 12/2017‐11/2022; Total $s (direct + indirect) = $506, 279/year for 5 years.
Discovery of novel therapeutic targets for IS and new target screening
**Swann laboratory**
*Publications* 1. Frost JD Jr, Le JT, Lee CL, et al Vigabatrin therapy implicates neocortical high frequency oscillations in an animal model of infantile spasms. Neurobiol Dis. 2015; 82:1‐11.
*Grant* NINDS NIH RO1; Infantile Spasms: Molecular Underpinnings of a Novel Combination Therapy: 3/2018‐2/2023; Total $s (direct + indirect) = $347,000/ year for 5 years.
*Patent* Patent published in October 2018. International publication number: WO 2018/195055 Al. PCT/US2018/027935. Title: Therapies for treating infantile spasms and other treatment‐resistant epilepsies.
**Galanopoulou laboratory**
*Publications* 1. Galanopoulou AS, Mowrey WB, Liu W, et al Preclinical Screening for Treatments for Infantile Spasms in the Multiple Hit Rat Model of Infantile Spasms: An Update. Neurochem Res. 2017; 42(7): 1949‐1961. 2. Galanopoulou AS, Mowrey WB, Liu W, et al Preclinical screening for treatments for infantile spasms in the multiple hit rat model of infantile spasms: an update. Neurochemical Res; 2017; 42(7): 1949‐1961. 3. Katsarou AM, Li, Q, Liu W, et al Acquired parvalbumin‐selective interneuronopathy in the multiple‐hit model of infantile spasms: a putative basis for the partial responsiveness to vigabatrin analogs? Epilepsia Open; 2018; 3 (S2): 155‐164. 4. Galanopoulou AS, Moshé SL. Pathogenesis and new candidate treatments for infantile spasms and early life epileptic encephalopathies: a view from preclinical studies. Neurobiology of Disease; 2015; 79:135‐149. 5. Shandra O, Moshé SL, Galanopoulou AS. Inflammation in epileptic encephalopathies. In Advances in Protein Chemistry and Structural Biology; 2017; 108:59‐84. 6. Katsarou AM, Moshé SL, Galanopoulou AS. Interneuronopathies and their role in early life epilepsies and neurodevelopmental disorders. Epilepsia Open; 2017; 2(3): 284‐306. 7. Barker‐Haliski ML, Loscher W, White HS, et al Neuroinflammation in epileptogenesis: insights and translational perspectives from new models of epilepsies. Epilepsia; 2017; 58(Suppl 3): 39‐47.
*Conference abstracts* 1. Brima T, Mowrey W, Moshé SL et al Efficacy and tolerability of an Interleukin 1 receptor antagonist (IL‐1Ra) in the multiple‐hit rat model of refractory infantile spasms. American Epilepsy Society Annual Meeting, Seattle Washington (2014). 2. Brima T, Mowrey W, Moshé SL, Galanopoulou AS. The efficacy of the mTOR inhibitor Torin1 on spasms in the multiple‐hit rat model of infantile spasms. American Epilepsy Society Annual Meeting, Philadelphia, PA, (2015). 3. Coles L, Cloyd J, Hovde L, et al Pharmacokinetics and Brain Uptake of IL‐1Ra in a Rat Model of Infantile Spasms. American Epilepsy Society Annual Meeting, Houston TX, (2016).
**Velíšek laboratory**
*Publications* 1. Chachua T, Di Grazia P, Chern CR, et al Estradiol does not affect spasms in the betamethasone‐NMDA rat model of infantile spasms. Epilepsia. 2016; 57(8): 1326‐1336. 2. Iacobas DA, Chachua T, Iacobas S, et al ACTH and PMX53 recover synaptic transcriptome alterations in a rat model of infantile spasms. Scientific Reports 2018; 8:5722. 3. Iacobas DA, Velíšek L. Regeneration of neurotransmission transcriptome in a model of epileptic encephalopathy after anti‐inflammatory treatment. Neural Regeneration Research 2018; 13:1715‐1718.
*Conference abstract* Iacobas DA, Chachua T, Iacobas S, et al Remodeling of synaptic transmission genomic fabrics in a model of infantile spasms. American Epilepsy Society Annual Meeting, Houston TX, (2016).
Discovery of biomarkers for IS
**Patel laboratory**
Publications 1. Heischmann S, Quinn K, Cruickshank‐Quinn C, Liang LP, Reisdorph R, Reisdorph N*, Patel M*. Exploratory metabolomics profiling in the kainic acid rat model reveals depletion of 25‐hydroxyvitamin D3 during epileptogenesis. Sci. Rep. 6:31 424; 2016. 2. Heischmann S, Gano LB, Quinn K, Liang LP, Klepacki J, Christians U, Reisdorph N, Patel M. Regulation of kynurenine metabolism by a ketogenic diet. J Lipid Res. 2018; 59(6): 958‐966. (*senior co‐corresponding authors.)
*Conference abstract* Heischmann S, Lee C, Le J, et al Plasma Metabolomic Analysis of (1‐3) IGF‐1 Treatment in Rats. Antiepileptic Drug Development (ADD) symposium, Utah (2015).
*Grant* 3R01 NS086423S1 Administrative supplement under FOA: Collaborative activities to promote metabolomics research. Direct costs: $118,598. Total funds: $164,987. Period: 7/2014‐6/2015.
Prognosis and treatment of IS
Sherr Team
*Publication* Yuskaitis CJ, Ruzhnikov MRZ, Howell KB, et al On behalf of the EPGP Investigators infantile spasms of unknown cause: predictors of outcome and genotype‐phenotype Correlation. Pediatr Neurol. 2018; 87:48‐56.

### 2.3 Execution of the IS initiative

Once research was underway, the PI representing each team attended quarterly meetings in which each investigator presented progress to the group for feedback. Investigators also submitted written progress reports annually.

These progress updates and discussions with advisors triggered real‐time shifting of scientific directions to work toward a common goal, in contrast to traditional academic research where investigators often proceed independently toward publication of results. Scientific steps were driven by outcomes of the research in a responsive manner. Decisions were often made following the principles employed by other disciplines such as those used in the pharmaceutical industry and were made by the advisory panel and CURE staff based on the overall scientific direction of the initiative, feasibility of next steps and budget. For example, if the investigators and the advisory panel believed that work in a particular model was not likely to lead to the identification of a potential therapy within defined timelines, the project was “sunsetted.” The decision to close out a program was not a judgment on the quality of the science, but a reality of a dynamic project where the scientific direction was driven by the outcomes. To this end, of the 8 initial projects, 2 projects were sunsetted partway through the initiative. The opportunity to change the direction of the science as the results of studies also inspired new scientific goals. For example, cross‐validation of potential targets in various animal models of IS was not predetermined, but its need arose to provide generalizability of findings as projects evolved. These decisions to change direction were communicated to investigators through discussions with CURE staff.

Ultimately, the goal of the initiative was to drive the understanding and potential treatments for IS forward utilizing the unique and diverse skill sets of the different teams in a collaborative manner.

## 3 RESULTS

### 3.1 Scientific outcomes of the IS initiative

The overarching scientific outcome of the IS initiative was an advancement in the phenotypical, electrophysiological, and pathophysiological understanding of IS, and in the utility of IS models for new therapy evaluation. In addition, several animal models of IS were tested and cross‐validated. Multiple compounds were tested, with one (insulin‐like growth factor‐1; IGF‐1) emerging as a promising therapeutic candidate. A metabolomics profiling workflow was established to analyze samples generated using the afore‐mentioned animal models. The structure of the IS initiative, and collaboration of the investigators with the advisory panel led to real‐time attrition of targets, rapid assessment of key targets and efficient exchange of scientific ideas. Overall, the IS initiative led to 19 publications, 7 publications in preparation (at the time of publication of this paper), additional funding [3 (Research Project Grant) RO1 grants], and a patent that was published in October 2018.^32^ Results are presented below based on following broad themes: (1) discovery on the basic biology of IS, (2) discovery of novel therapeutic targets for IS and new target screening, (3) cross‐validation of targets, (4) discovery of biomarkers for IS, and (5) prognosis and treatment of IS. A list of publications, abstracts, grants, external funding, and patents acquired as a result of the IS initiative are listed in Table 1.

#### 3.1.1 Discovery on the basic biology of IS

A number of projects within the initiative focused on understanding the basic biology underlying IS. The Noebels laboratory studied the effects of hormones in the complex neurological phenotype of IS in light of previous research showing that 17β‐estradiol (E2) when given during an early postnatal (P3‐10) period, but not in the adult period, prevents the clinical phenotype of spasms, spikes and seizures.^33^ A number of compounds and therapeutic approaches were tested to extend these findings. Experiments in the Arx^(GCG)10+7^ model revealed that delayed treatment with E2 and doubling the dose in the second neonatal week following onset was less protective against subsequent seizures than shown in previous studies.^34^ One tested dose of a selective estrogen β receptor agonist, LY500307, displayed protective effects, and a brain‐selective E2 prodrug that is converted to E2 in brain appeared as effective as systemic E2. ACTH did not prevent spasms in the Arx^(GCG)10+7^ model, but did trend toward reducing inter‐ictal spike frequency at one dose tested.

In addition to testing the effects of steroids, several mechanistic pathways were explored in the Arx^(GCG)10+7^model. An early and transient wave of programmed cell death that normally occurs in the first postnatal week was abnormally elevated in Arx^(GCG)10+7^ mutant mice, yet this was not due to excitotoxicity or an innate immune response, since there was no cellular evidence of microglial inflammation and no abnormal transcriptional signaling indicating molecular inflammatory activity.^35^ E2 did not affect the augmented apoptosis suggesting that the underlying mechanism was neuroprotective rather than anti‐inflammatory, and that enhanced apoptosis in the Arx^(GCG)10 +7^ model was not solely responsible for the seizure phenotype, but may contribute to other brain deficits in the Arx^(GCG)10+7^ model.^35^ In collaboration with the laboratory of Mauro Costa‐DiMatteoli at Baylor College of Medicine, the Noebels laboratory made the discovery that activation of the mechanistic Target of Rapamycin (mTOR) complex 2 (mTORC2), rather than mTORC1, is responsible for the antiepileptic effects of rapamycin therapy, and its reduction could represent a promising translational target for early brain disorders associated with IS where mTOR is dysregulated, such as tuberous sclerosis.^36^

The Dulla laboratory studied conditional loss of APC in excitatory neurons, and the role of ß‐catenin and aberrant Wingless‐related integration site (Wnt) and synaptogenic protein networks in synaptic and circuit dysfunction associated with IS. Experiments revealed feasibility of the APC cKO model as a new mouse model of IS.^37^ Moreover, as a result of a collaboration with the Noebels laboratory that allowed for the transfer of video‐EEG techniques, the Dulla laboratory developed preliminary data investigating the role of ß ‐catenin in the Arx^(GCG)10+7^ model. NIH funding to continue work on the role of ß‐catenin in IS was obtained,^38^ and the Dulla laboratory continues to investigate the role of synaptic and developmental changes that underlie the potential connection between ß‐catenin and IS. A list of publications, abstracts, grants, external funding, and patent acquired as a result of the IS initiative are shown in Table 2.

#### 3.1.2 Discovery of novel therapeutic targets for IS and new target screening

The Swann laboratory investigated specific targets and novel therapeutic strategies to stop spasms and ameliorate underlying neurodevelopmental abnormalities. The team had shown that expression of IGF‐1 in the neocortex, PI3K‐AKT‐mTOR signaling, and expression of parvalbumin and synaptotagmin 2 are suppressed in a model of IS.^39^ Carrying these results forward, they showed that treatment of a TTX rat model with (1‐3)IGF‐1—a tripeptide derivative from IGF‐1—rescued inhibitory interneurons and abolished spasms and Hypsarhythmia. When tested with vigabatrin, a standard of care for IS, (1‐3)IGF‐1 induced a synergistic effect with vigabatrin allowing for a reduction in the dosage of vigabatrin required to abolish spasms. Retinotoxicity, specifically, irreversible peripheral visual field constriction, has been reported with vigabatrin. Additional funding was provided for pharmacokinetic and toxicity studies to explore this issue. Experiments showed that indeed, vigabatrin retinotoxicity was greatly reduced when it was combined with the naturally occurring tripeptide, (1‐3)IGF‐1. Additionally, the pharmacokinetics of vigabatrin were unaltered by (1‐3) IGF‐1. Experiments also revealed that combination treatment was effective when (1‐3)IGF‐1 was given orally.

The Galanopoulou laboratory proposed to identify new therapeutic targets and treatments for IS using treatment protocols tailored by pharmacokinetic modeling done in the Coles laboratory and tested in vivo in the multiple‐hit rat model. For example, they showed a therapeutic advantage of intracerebrally administered anakinra (Kineret®), which is a Food and Drug Administration (FDA)‐approved immunosuppressant, on various epilepsy and neurodevelopmental endpoints. Preliminary data showed that as compared to vehicle, anakinra demonstrated greater control of spasms, improvement of neurodevelopmental milestones, visuospatial learning and memory in the Barnes maze, and sociability. The team also interrogated the therapeutic benefits of compounds that inhibit the mTOR pathway. Based on prior studies that demonstrated spasm cessation and partial disease modification after pulse treatments with high doses of rapamycin—an mTORC1 inhibitor,^40^ studies were designed to investigate the effects of dual mTORC1/mTORC2 inhibitor torin 1.^41^ Additionally, in an attempt to cross‐validate compounds, targets from the Noebels laboratory were investigated in the Galanopoulou laboratory. These experiments showed a lack of protective effect of E2 in the multiple‐hit model;^42^ more details can be found in section (3) cross‐validation of targets.

The Velisek laboratory explored the effect of E2 treatment in a convulsant‐induced model of IS where neonatal rats were prenatally exposed to betamethasone on day 15 of pregnancy and later exposed to NMDA.^43^ While efficacy of E2 in this model was not observed, they observed a significant increase in glutamic acid decarboxylase 67 (GAD67)‐immunopositive cells in the neocortex of the immature brain following treatment.^44^ The work then shifted to the investigation of a potential treatment target among the inflammatory molecules. The group focused on the complement C5a receptor 1 antagonist PMX53. They found that besides significant suppression of spasms by PMX53 comparable to that by ACTH, after the treatment there was also a recovery in the gene profiles of genes governing synaptic transmission.^44^ This finding suggests a possible biomarker for efficacy of treatment in IS.^45^ The project was discontinued as part of the overall IS initiative after a year. However, the laboratory continued to pursue new effective treatments for IS with fewer adverse effects and has been further supported by the biopharma industry.^46^, ^47^

#### 3.1.3 Cross‐validation of targets

The structure of the initiative allowed for cross‐testing of promising targets in additional models. E2 did not have a protective effect in the multiple‐hit model or the prenatal bethametasone‐postnatal NMDA model,^42^, ^43^ and the anti‐seizure effects of (1‐3)IGF‐1 were not reproduced in the other models tested. The reason for this may be related to the differences in the models used. Acute models include the NMDA model, either on its own or with prior prenatal betamethasone or perinatal stress exposure, and the γ‐butyrolactone‐induced spasms in a mouse model of Down syndrome.^27^, ^28^, ^29^, ^30^, ^31^ Chronic models include the TTX rat model, the Arx^(GCG)10+7^ mouse models and the multiple‐hit rat model. Commonalities between the models include interneuronopathies and the mTOR pathway, but differences abound; and the lack of effect of E2 and (1‐3)IGF‐1 in the additional models tested is not surprising given these differences.^48^ Additional studies are needed to better understand if the targets such as E2 and (1‐3)IGF‐1 are effective in certain forms of IS and not others. Since this was a team initiative, cross‐validation of targets was possible, and execution of cross‐validation was much smoother than had the investigators been individually funded.

#### 3.1.4 Discovery of biomarkers for IS

The Patel laboratory studied the metabolome with a goal of uncovering potential biomarkers and targets for IS. An unbiased metabolomics profiling workflow was established for analysis of plasma and brain tissue samples, and experiments were designed to prove applicability of the workflow to analyze matrices relevant to the scope of the broader initiative ^34^, ^49^ and ultimately provide a better idea of the metabolic changes underlying IS (publications in preparation). In addition, there were collaborations with the Noebels laboratory to characterize the Arx^(GCG)10+7^ genotyped mice, the Galanopoulou laboratory to explore metabolomics in the multiple‐hit model and anakinra treatment model, the Swann laboratory to study the effects of (1‐3)IGF‐1 treatment in male and female rats, and the Dulla laboratory to characterize APC cKO mouse brain and plasma samples.

Dependent on the respective needs of the collaborating laboratories, protocols for sample collection and processing were established at the Patel laboratory. Factors to be taken into account were, for example, the sample type (eg, specific brain region vs. whole brain, collection of trunk blood vs. blood draw by cardiac puncture) and availability of sufficient material (eg, in the case of collection of brains of mice on postnatal day 9). Preliminary metabolomic analysis of plasma of (1‐3)IGF‐1‐treated rats indicated that it is crucial to distinguish between male and female populations when examining the effects of (1‐3)IGF‐1 treatment. In addition, results of this preliminary study indicated that pathways related to endocannabinoid signaling and disruptions in ACTH metabolism among others may be targets of the treatment (publication in preparation).

The Nordli team studied whether an EEG signature of pre‐Hypsarhythmia could be identified that would predict the onset of IS as well as whether ACTH would improve EEG abnormalities in infants with pre‐Hypsarhythmia. The team recruited 44 human subjects for an EEG study and performed data cleaning and classification on all subjects. Two subjects with pre‐Hypsarhythmia were treated under a separate Institutional Review Board (IRB) protocol with low dose ACTH, and subsequently were treated under FDA‐approved ACTH treatment protocol. While preliminary, these studies supported the use of pre‐Hypsarhythmia as a biomarker in children at risk for developing IS.

#### 3.1.5 Prognosis and treatment and of IS

The Sherr team investigated “infantile spasms of unknown cause” (UCIS)—a subset of IS patients with better overall outcome and potential of improved long‐term prognosis by effective treatment of spasms. The primary aim was to investigate variables such as Hypsarhythmia and response to treatment associated with UCIS. An additional aim was to specify genomic variants associated with the neurodevelopmental and neuropsychiatric abnormalities, and epilepsy syndromes in UCIS. The team studied a large cohort of UCIS patients, showing that there was a strong correlation between having a defined genetic diagnosis and a less favorable developmental outcome. Moreover, only 15% of the cohort had a favorable developmental outcome (unpublished data). Predictors of positive developmental outcomes included no delay prior to IS, older age of IS onset, and resolution of IS after initial treatment. However, the project was discontinued as experiments did not address development of a treatment.

### 3.2 Cultural outcomes of the IS initiative

The testimonies of advisors and investigators on how the initiative affected their work ranged from gaining a greater appreciation of the role of patient organizations for epilepsy drug discovery, to the realization of steps involved in taking targets through clinical research, and the value of collaboration. For example, one advisor said “I think it was a great example of how researchers can collaborate for a common scope and put their energies toward a better understanding of disease causes and therapies with a defined molecular action. I think that without this opportunity to work together these researchers would have been competing rather than collaborating on a common ground, and this collaboration is feasible and fostered only in the context of a common grant.” One investigator mentioned “what was different about the IS initiative was getting basic scientists and clinicians together for intense exchanges of information and ideas. Although I work at a medical center, that does not happen that often. Moreover, the clinicians did not always agree and that was informative.” Another investigator agreed and stated that a major advantage was the rapid transfer of information between teams and faster cross‐validation of results in some cases, rather than waiting to learn of results following publication. “Time is our most precious asset, and any consortium arrangement that can accelerate therapeutic trials, win or lose, is an enormous advantage to the patients and families we hope to benefit.”

Mentoring of junior scientists was a key part of the IS initiative, and a total of 6 junior mentees were funded. These junior investigators benefited from partnering with established scientists and exposure to research findings and working practices from other PIs.

The initiative fostered additional active collaborations among several team members. “Cross fertilization between clinicians and basic scientists was especially valuable for both groups, which do not always have a comfort zone for critical discussion.” According to one investigator, “Not only did it inspire us to think about novel treatments, but also new ways to prevent the development of this catastrophic epilepsy in the first place.”

Areas of growth were related to the practicalities of an initiative of this nature. One issue was that only one person from each team, usually the PI, could have a voice, limiting input from other members of the teams. This was an important learning from this initiative and speaks to the importance of bringing multiple team members to the table from the outset of any team project. Another issue was that “Occasionally, though infrequently, team priorities superseded the laboratory priorities and shifted direction of research in some laboratories to directions that investigators would not have pursued and could not follow up or utilize at the conclusion of funding.” One example of this was the cross‐validation of targets in multiple animal models, that while critical in development of new therapeutics, was less advantageous for academic laboratories that need to pursue innovative and novel science.

The biggest impacts of the initiative included the following: acceleration of findings in the field of IS, bolstering of collaboration, and cross‐validation of targets. Experience gained from this initiative can be applied to subsequent projects related to epilepsy, and to other disorders where transformative therapies are needed. The framework of the initiative enabled investigators to trust each other with confidential information, which would have been difficult in a traditional academic setting. Ultimately, the initiative enabled researchers who normally would not have collaborated with each other to join forces for a common cause.

### 3.3 Learnings

The intent of the IS initiative was to have investigators collaborate rather than compete with one another. Traditional academic science can take many years from submitting a grant to publication of results, but in the IS initiative data and resources were shared in real‐time. The inclusion of the advisory panel was critical to ensure that PIs shared findings with the group in a transparent manner. A major lesson was related to project management. Inclusion of a full‐time project manager could have helped streamline scheduling and processes such as the collection of reports. While there were no authorship concerns in this project, any initiative would benefit from a well‐defined and transparent publication and authorship policy. Learnings from the CURE IS initiative have been implemented in CURE’s ongoing Post‐Traumatic Epilepsy (PTE) initiative, wherein a project manager oversees daily activities, resolves issues, schedules meetings well in advance and develops policies that set expectations on confidentiality, publication and data‐sharing.

Although one of the targets [(1‐3)IGF‐1] revealed as a result of efforts of the IS initiative gained attention and resources, the same could not be said of other, perhaps equally promising, targets. Hence, another lesson was that criteria for “success” of a project as well as next steps once a target is defined should have been predetermined and clearly communicated. Similarly, additional expertise in the fields of pharmacokinetics, drug dosing, drug formulation, biostatistics, clinical trials or design could have been sought in advance. This initiative highlighted the challenges of moving drugs from the preclinical to the clinical setting. A related point is that the initiative would have benefitted from consistent clinical insight. Engaging a clinician to advise on every step of the project may have led more translatable findings. Also, leveraging findings from preclinical studies, and pre‐defined criteria for entry of a target into the clinic would have helped guide processes more efficiently. Whether (1‐3)IGF‐1 becomes a transformative therapy for IS treatment will have to await the results of a clinical trial. As of 2020, this target has not advanced beyond preclinical testing.

## 4 CONCLUSION

Team science initiatives have gained traction in recent years in an effort to address complex healthcare questions.^50^ CURE has broad expertise in funding investigator‐initiated research projects and collaborative, multi‐disciplinary grant programs. CURE took on the challenge to advance the understanding of and discover potential new treatments for IS through this team science approach. The IS initiative brought together a diverse team of experts to rapidly advance IS research and was the first such initiative in the field of epilepsy. Advantages of the IS initiative included an accelerated timeline for discoveries, increased collaboration between research teams, and facilitation of cross‐validation of targets. Learnings from the IS initiative are being used for CURE’s PTE initiative.

## CONFLICTS OF INTEREST

AS Galanopoulou is Editor in Chief of *Epilepsia Open* and has received royalties for publications from Elsevier and Morgan and Claypool publishers. H Klitgaard was an employee of UCB Pharma during the period of the research activity. JJ Millichap reports personal fees from American Academy of Neurology, personal fees from Up‐To‐Date, grants and personal fees from UCB Pharma, grants and personal fees from Mallinkrodt, personal fees from Esai, grants and personal fees from Xenon, personal fees from Biomarin, personal fees from Ionis, personal fees from Greenwich, personal fees from Sunovion, personal fees from Upsher‐Smith, grants from NIH, grants from Citizens United for Research in Epilepsy, personal fees from Praxis, grants and personal fees from Disruptive Nutrition, personal fees from Sarepta, outside the submitted work. SL Moshé is serving as Associate Editor of *Neurobiology of Disease* and is on the editorial board of *Brain and Development, Pediatric Neurology* and *Physiological Research*. He receives from Elsevier an annual compensation for his work as Associate Editor in *Neurobiology of Disease* and royalties from 2 books he co‐edited. He has received consultant's fees from UCB and Pfizer. A. Pirone is currently an employee at Alkermes. J Rho is a member of the scientific advisory boards for Scientific Advisory Boards for The Charlie Foundation (Santa Monica, CA), Matthew's Friends Foundation (Surrey, United Kingdom), and consultant for Danone Nutricia, Accera, Inc, Cypralis, Ltd., Ajinomoto USA, UCB Pharmaceuticals, Eisai Pharmaceuticals, Mallinckrodt Pharmaceuticals, Aquestive Pharmaceuticals. A Vezzani has served as a paid consultant for Biogen, Cambridge, MA, USA and has received research support from UCB Inc, Brussels, Belgium. None of the other authors has conflicts of interest to disclose. The authors confirm that we have read the Journal's position on issues involved in ethical publication and affirm that this report is consistent with those guidelines.

## Appendix 1

*Advisors*: Annamaria Vezzani (Mario Negri Institute for Pharmacological Research, IRCCS, Milano, Italy); Anne T. Berg (Northwestern University Feinberg School of Medicine, Chicago, IL, USA); Daniel H. Lowenstein (University of California, San Francisco, CA, USA); Henrik Klitgaard (UCB Pharma, Neurosciences Therapeutic Area, Braine l'Alleud, Belgium); Howard P. Goodkin (University of Virginia School of Medicine, Charlottesville, VA, USA); Jong M. Rho (University of California San Diego, San Diego, CA, USA).

*Investigators*: Anna Maria Katsarou (Albert Einstein College of Medicine, Bronx, New York, NY, USA); Antonella Pirone (Tufts University School of Medicine, Boston, MA, USA); Aristea S. Galanopoulou (Albert Einstein College of Medicine, Bronx, New York, NY, USA); Chris Dulla (Tufts University School of Medicine, Boston, MA, USA); Douglas R. Nordli (The University of Chicago, Chicago, Illinois, USA); Dumitru A. Iacobas (Prairie View A&M University, Personalized Genomics Laboratory, Prairie View, TX, USA); Elliott Sherr (University of California San Francisco, San Francisco, CA, USA); James Cloyd III (University of Minnesota Center for Orphan Drug Research, Minneapolis, MN, USA); Jana Veliskova (New York Medical College, Valhalla, NY, USA); Jeff L. Noebels (Baylor College of Medicine, Houston, TX, USA); John J. Millichap (Northwestern University Feinberg School of Medicine, Chicago, IL, USA); John W. Swann (Baylor College of Medicine, Houston, TX, USA); Sookyung Koh (Northwestern University Feinberg School of Medicine, Chicago, IL, USA); Libor Velisek (New York Medical College, Valhalla, NY, USA); Lisa Coles (University of Minnesota Center for Orphan Drug Research, Minneapolis, MN, USA); Manisha Patel (University of Colorado, Aurora, CO, USA); Meagan S. Siehr (Baylor College of Medicine, Houston, TX, USA); Michele Jacob (Tufts University School of Medicine, Boston, MA, USA); Ryan Seo (Baylor College of Medicine, Houston, TX, USA); Svenja Heischmann (University of Colorado, Aurora, CO, USA); Solomon L. Moshe (Albert Einstein College of Medicine, Bronx, New York, NY, USA); Tamar Chachua (New York Medical College, Valhalla, NY, USA); Tufikameni Brima (Albert Einstein College of Medicine, Bronx, New York, NY, USA); Wenzhu B. Mowrey (Albert Einstein College of Medicine, Bronx, New York, NY, USA).

*CURE Staff*: H. Steve White, Julie Milder.
